# Optimizing sentinel surveillance in temporal network epidemiology

**DOI:** 10.1038/s41598-017-03868-6

**Published:** 2017-07-06

**Authors:** Yuan Bai, Bo Yang, Lijuan Lin, Jose L. Herrera, Zhanwei Du, Petter Holme

**Affiliations:** 1 College of Computer Science and Technology, Jilin University, Changchun, 130012 China; 2 Key Laboratory of Symbolic Computation and Knowledge Engineering of Ministry of Education, Jilin University, Changchun, 130012 China; 3 Department of Integrative Biology, University of Texas at Austin, Austin, 78705 United States; 4 ICTP South American Institute for Fundamental Research, Sao Paulo State University, Sao Paulo, 03001-000 Brazil; 5 Institute of Innovative Research, Tokyo Institute of Technology, 152-8550 Tokyo, Japan

## Abstract

To help health policy makers gain response time to mitigate infectious disease threats, it is essential to have an efficient epidemic surveillance. One common method of disease surveillance is to carefully select nodes (sentinels, or sensors) in the network to report outbreaks. One would like to choose sentinels so that they discover the outbreak as early as possible. The optimal choice of sentinels depends on the network structure. Studies have addressed this problem for static networks, but this is a first step study to explore designing surveillance systems for early detection on temporal networks. This paper is based on the idea that vaccination strategies can serve as a method to identify sentinels. The vaccination problem is a related question that is much more  well studied for temporal networks. To assess the ability to detect epidemic outbreaks early, we calculate the time difference (lead time) between the surveillance set and whole population in reaching 1% prevalence. We find that the optimal selection of sentinels depends on both the network’s temporal structures and the infection probability of the disease. We find that, for a mild infectious disease (low infection probability) on a temporal network in relation to potential disease spreading (the *Prostitution* network), the strategy of selecting latest contacts of random individuals provide the most amount of lead time. And for a more uniform, synthetic network with community structure the strategy of selecting frequent contacts of random individuals provide the most amount of lead time.

## Introduction

Despite the medical advances, the outbreak of infectious disease is a persistent major burden to human health. For example, in 2009 the H1N1 influenza epidemic spread across 214 countries, causing an estimated economic loss of USD 3 trillion (nearly 5% of the global GDP) and leaving a death toll of 18,500^1–4^. More recently, the Zika virus outbreak—that started in the South and Central America and the Caribbean in 2015—is expected to cost an estimated USD 3.5 billion in these regions and lead to more than USD 1.5 million infections around the world in 2016^5–8^. To reduce human suffering and economic loss, it is important to discover epidemic outbreaks early. For this purpose, health agencies have designed surveillance systems. These systems not only help to detect outbreak, but also help to forecast the future extent and duration of the outbreak^9–13^. The most important property of such systems is to identify an emerging outbreak as early as possible. It is a similar problem to network vaccination where the task is to find optimal individuals to vaccinate (or otherwise incapacitate with respect to the disease spreading). This problem is somewhat more well-studied in contact of temporal networks^14–16^. Optimizing the sentinel selection, however, puts an emphasis on early detection and is not concerned with the expected number of others that can be infected in an outbreak starting from a certain individual.

Surveillance strategies, which have been used in surveillance systems for early detection, fall into two general categories—those based on monitoring care-seeking behavior and those mapping out contact behavior. Surveillance systems based on the first category strategies try to monitor individuals who seek outpatient care in sentinel hospitals or online search engines and calculate the number of cases among those individuals. For example, some traditional surveillance systems, such as the Hong Kong Centers for Health Protection (HP) and the United Kingdom Health Protection Agency (HPA), are based on hospital systems. They monitor individuals who see a doctor in sentinel hospitals and report their incidence of infectious disease for early detection^17^. Some other surveillance systems, such as (currently defunct) Google Flu Trends, are based search engines queries. Such systems monitor users who search for health information online and provide real-time information on disease outbreaks^17–22^.

In contrast to care-seeking surveillance systems, the second contact-behavior category tries to monitor a set of individuals who can catch and transmit the disease much earlier than the average individual. Previous research has designed surveillance strategies for early detection of infectious disease based on topological structures of static contact networks. For example, ref. 23 presented a simple surveillance strategy monitoring friends of randomly selected individuals. They also use this method to detect the spread of flu at Harvard College. The epidemic situation in this strategy occurred about two weeks earlier than the strategy which selects individuals randomly to monitor. Moreover, to find the suitable scenarios for these network-oriented strategies, ref. 11 compared three surveillance strategies (selecting individuals with the highest degree, individuals randomly and friends of random individuals) on three classes of static contact networks (networks with power-law degree distribution, Poisson degree distribution and community structure). They found the optimal strategy depends on the network structures and the basic reproduction number of the disease. If a disease has a low basic reproduction number, the strategy of selecting individuals with the highest degree would provide the earliest information about both the onset and peak of an outbreak. However, if there is no prior knowledge of the network, a practical alternative is selecting friends of random individuals. Surveillance systems based on care seeking can identify an outbreak one or two weeks after the onset or peak of an outbreak or contemporaneously at best. Hypothetically, surveillance systems based on contact-behavior strategies could improve on this. So far such strategies have neglected the fact that contacts are not static^24, 25^. Contact networks not only have topological structures, but also temporal structures (e.g., cyclic ones—an individual meeting a friend on a daily basis)^14^.

The temporal structures of networks not only affect the speed of spread and the final outbreak size but also strategies to mitigate the outbreak^16, 25–28^. Indeed, the earlier studies have shown that vaccination strategies that exploit temporal structures outperform the neighborhood vaccination strategy to effectively control infectious disease^14–16^. To our knowledge, surveillance strategies that exploit temporal structures to select sentinels for early detection of infectious disease have not been investigated before. That is the goal of this paper.

In this study, we investigate whether vaccination strategies designed for static or temporal networks can serve as suitable strategies for selecting sentinel nodes. To address this problem, we compare two temporal-network strategies for selecting sentinels (sampling the last contact and most frequent contact with random individuals) with two static-network strategies (sampling random contact of random individuals and random individuals) on four temporal networks under a well-studied epidemic scenario (discussed below). Specifically, we first divide the time span of a temporal network into two time windows. In addition, we select sentinels based on different surveillance strategies in the first time window. Finally, we simulate the susceptible-infected (SI) epidemic model to evaluate different surveillance strategies based on lead time in the second time window. We test the efficacy of the surveillance strategies by measuring how long sampling the sentinels increases the lead time in epidemic simulations. The SI model models diseases with relatively long infection times compared to the dynamics of the outbreak.

## Materials and Methods

### Temporal network data set

In this section, we will present the temporal networks we use as models for the underlying network structure. We will discuss the nodes in this network as individual people. Most of the empirical networks we use are indeed networks of people. In practice, sentinels would rather be healthcare units (wards, clinics, hospitals), or farms (in the case of disease among livestock).

Our first network, *Prostitution*, was collected from a Brazilian online forum. This dataset includes *N* = 16,730 individuals and *E* = 50,632 sexual contacts spanning *T* = 2,232 days. The contact created at time *t* between a pair of individuals in the dataset represents the reported sexual encounter between a male sex-buyer and a female escort^14, 29^. The *Dating* network comes from an Internet dating community^30^. This dataset includes *N* = 28,972 individuals and *E* = 430,827 sexual contacts and spans *T* = 512 days. Contacts represent the initial interaction between two people (with the intention to pursue a future off-line relationship)^14, 30^. The *E*-*mail* data was collected from one of the main mail servers of a university^31^. This dataset includes *N* = 2,997 email accounts and *E* = 202,694 contacts and spans *T* = 82 days. The contact created at time *t* between a pair of email accounts in the dataset represents one email address sending or receiving a message at a particular time^14, 31^. In addition, we use a *Synthetic* model to generate the large-scale temporal networks with community structure to compare with the *Prostitution* and *Dating* networks. To generate this data, we use the following approach: First, we use the Lancichinetti-Fortunato-Radicchi model to generate a scale-free static network with community structure^32^. Then, we add time stamps for every edge in the network generated by the above step based on the varying activity model in refs 14, 33. Since this network model algorithm is stochastic, we average all values over five realizations of the algorithm. This dataset includes *N* = 20,000 nodes and average *E* = 1,186,200 edges and spans *T* = 2,000 time steps. The specific parameters we use in this construction are shown in Table 1.

**Table 1:** Detailed information about the synthetic network.

Parameters	Meaning	Value
*N*	number of nodes	20000
〈*k*〉	average degree	15
*k*_max_	maximal degree	50
*μ*	mixing parameter	0.3
*c*_min_	minimum community size	100
*c*_max_	maximum community size	1000
*λ*_1_	the exponents of the power law distributions of the node degree	2
*λ*_2_	the exponents of the power law distributions of the community size	1

**Figure 1:**
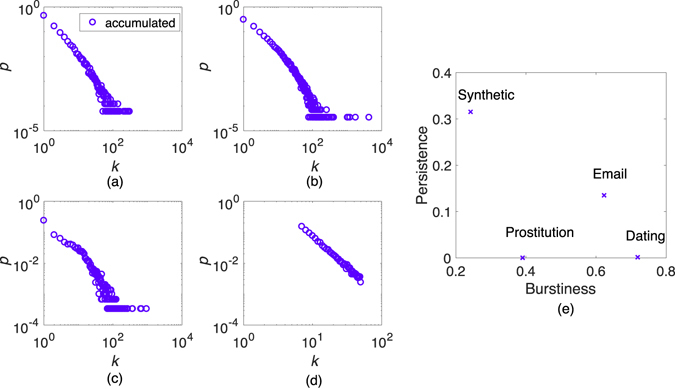
The statistical characteristics of the temporal networks. In panels (a–d), we plot the probability density *p* as a function of degree *k* ((**a**) *Prostitution* network, (**b**) *Dating* network, (**c**) *Email* network and (**d**) *Synthetic* network). We plot results for the accumulated network of all contacts. In panel (e), we show the temporal statistics of the datasets—persistence and burstiness.

We summarize some properties of our temporal networks in Table 2 and Fig. 1. The *Synthetic* entries in Table 2 and Fig. 1 show the average properties of the *Synthetic* model networks. Regarding the static network structure, the degree distributions of networks of accumulated contacts are qualitatively similar for all four datasets (see Fig. 1(a–d)). Regarding temporal structures, the four datasets all show a high burstiness (the phenomenon that human activities often happen in intense periods separated by periods of quiescence^14^, see SI text). However, they have different persistence (measured by the fraction of edges that is present both in the first and last 5% of the contacts by the Jaccard similarity coefficient^14^). The *Prostitution* and *Dating* networks are the most dynamic, in the sense that individuals are entering and leaving the system throughout the sampling period. The *Email* network is less dynamic than the first two networks. The *Synthetic* is by construction the most uniform in terms of activity. At the same time, this network exhibits not only a broad degree distribution structure, but also obvious community structure (similar as the other three networks).

**Table 2:** Some properties of temporal networks.

Dataset	*N*	*E*	*T*	Δ*t*	〈*k*〉	*M*
*Prostitution*	16730	50632	2232d	1d	15	0.682
*Dating*	28972	430827	512d	1s	8	0.448
*Email*	2997	202694	82d	1s	15	0.406
*Synthetic*	20000	1186200	2000 time steps	1 step	15	0.670

Number *N* of different individuals generated *E* edges during *T* time steps. Δ*t* represents time units, 〈*k*〉 represents average degree, *M* represents modularity of the accumulated networks.

### Surveillance strategies

A surveillance strategy is a method to select a surveillance set *υ* of sentinels. Here, we focus on two temporal-network surveillance strategies which refer to the immunization strategies on temporal networks^14^ and two static-network strategies. As mentioned, we divide the time duration of individual contacts *T* into two parts [0, 3*T*/4] and [3*T*/4, *T*] (Our results are roughly speaking stable in the interval *T*/2 < t* < 4*T*/5 when the fraction of individuals in the surveillance set ranges from 10% to 12%, here we settle for t* = 3*T*/4). The time window [0, 3*T*/4] is the experience period. We can use the information in this period as a guidance to the sentinel assignment. Then we use the later time window, [3*T*/4, *T*], for disease simulation and evaluation of the surveillance strategies. This *ex ante* methodology circumvents the unreasonable assumption of static network modeling that the past accurately predicts the future (i.e., that the network remains unchanged).

Another important issue is that it is infeasible to assume a complete knowledge of the network even during the experience period. Mapping out a social network is costly, time-consuming and inaccurate^34, 35^. At best, one can hope to get local information of the network in the neighborhood of some specific interviewees. For this reason, we consider only such local strategies and omit strategies based on the knowledge of the entire (or a large part of) the network. In the spirit of ref. 36, three of our strategies proceed by first inquiring a randomly selected individual about his or her contacts, and base the sentinel selection on that information. Our fourth strategy is a completely random reference strategy.

**Figure 2:**
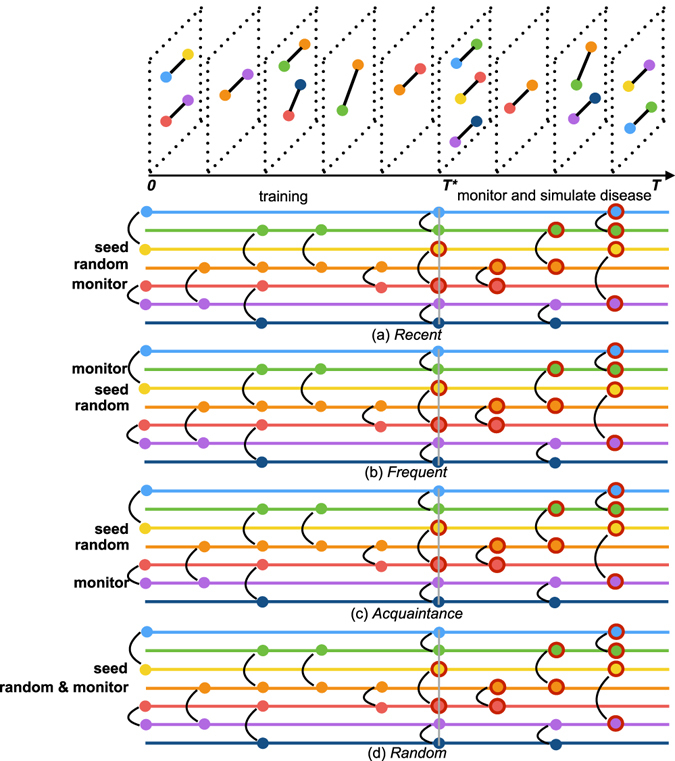
An illustration of our surveillance strategies. The top diagram illustrates a temporal network of contacts over a time window. There are two time windows, separated at three quarters(round down) of the sampling time, one for training and one for disease spreading simulation. In Panels (a–d), each horizontal line represents an individual and the circles and vertical lines indicate the interaction. The colors of the lines are the same as the vertex colors. The first infected node is marked “seed” and the interviewee selected randomly for information gathering is marked “random”. The sentinel represents selected neighbor of the interviewee selected according to different strategies. In the *Acquaintance* strategy, the orange red and green nodes can also be selected as sentinels. In the time window for disease spreading, red circle marks infected individual. The epidemic model in this example is the SI model with 100% infection probability.

Our four strategies work as follows (also see Fig. 2):

#### Recent

We select an individual randomly and add the most recent contact in [0, 3*T*/4] to *υ*. We repeat this process until we reach a fraction *f* of individuals in the surveillance set.

#### Frequent

We select an individual randomly and monitor his or her most frequently contacted neighbor in [0, 3*T*/4]. This process is then repeated until we have added a fraction *f* of individuals to *υ*.

#### Acquaintance

We select an individual randomly and a random neighbor (throughout the experience period [0, 3*T*/4]). We repeat this process until a fraction *f* of individuals belong to *υ*.

#### Random

We let *υ* be a random fraction *f* of individuals present in [0, 3*T*/4].

### Disease-spreading model and lead time

We simulate the dynamics of epidemic spreading on temporal networks using the simplest compartmental model—the susceptible-infected (SI) model. Every individual is susceptible to the disease to start with. Upon meeting an infected individual a susceptible node can become infected. Even though this is an extreme situation (in particular with a 100% infection probability), it can provide interesting insights into the role of temporal and topological structure for disease spreading^15^. In this work, we study the SI model on temporal networks in time window [3*T*/4, *T*]. The infection is assumed to originate from one source randomly at time 3*T*/4. At each time step, each susceptible individual in contact with an infected will become infected with the disease’s per-contact infection probability *β*. The propagation process will stop when there are no susceptible individuals or the contact time series is exhausted.

**Figure 3:**
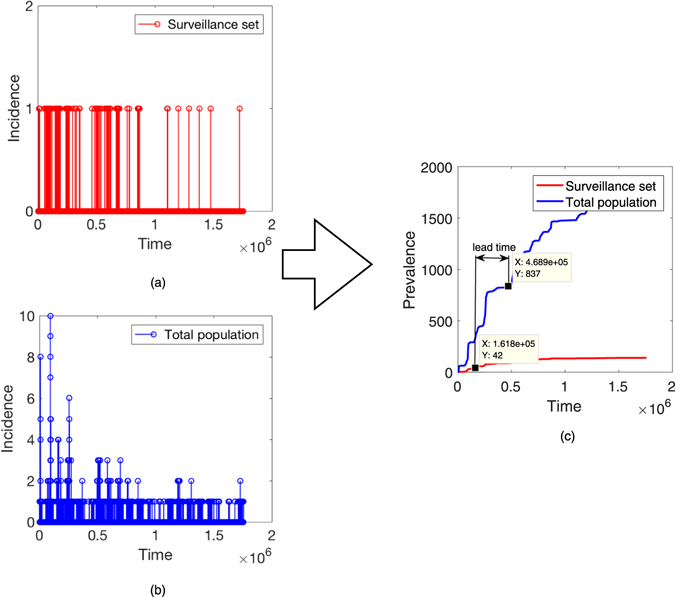
An illustration of an epidemic. In panels (a,b), we see incidence in surveillance set and total population respectively. In panel (c), we calculate the lead time between the surveillance set and total population reaching 1% prevalence.

The insight from vaccination studies is that during an epidemic, some individuals could be more efficient spreaders than others. It would be no surprise if this property is correlated with the aptitude of being a sentinel. Fig. 3 gives an illustration of a disease simulation run. To estimate this impact, we ran SI model several times and calculated the average lead time *ω*_*υ*_

1 $${\omega }_{\upsilon }=\langle {T}_{\rho }-{T}_{\upsilon }\rangle ,$$

where *T*_*ρ*_ is the time point at which the outbreak have reached 1% prevalence in the whole population and *T*_*υ*_ is the time at which the prevalence within the surveillance set is 1%. This is about the percentage needed to infer an outbreak from sentinel surveillance^11, 37, 38^. A larger *ω*_*υ*_ means that we have more time to respond and fight infection. Note that, first, if the individuals in the surveillance set *υ* are not infected during the time window [3*T*/4, *T*] in which the SI is simulated, then we set *T*_*ρ*_ = *T*_*υ*_. Second, if the prevalence in the surveillance set *υ* does not reach 1% prevalence, then we set *T*_*ρ*_ = *T*_*υ*_. Third, when *T*_*ρ*_ < *T*_*υ*_, we set *T*_*ρ*_ = *T*_*υ*_.

## Results

Now we will turn to analyzing our numerical results.

### Surveillance strategies evaluation

**Figure 4:**
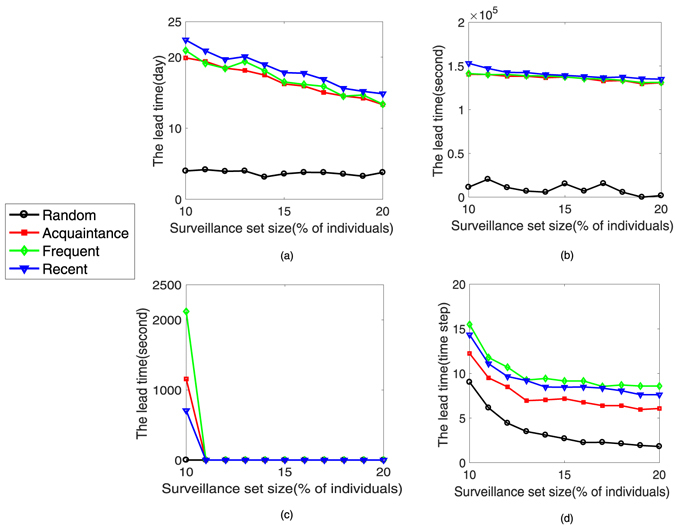
The size of surveillance set impacts performance. How the lead time improves as the surveillance set expands in the (**a**) *Prostitution* network, (**b**) *Dating* network, (**c**) *Email* network and (**d**) *Synthetic* network. The surveillance set was chosen by the *Random* (black), *Acquaintance* (red), *Frequent* (green) and *Recent* (blue) strategies.

To evaluate our four surveillance strategies (two temporal-network surveillance strategies and two static-network strategies) on the four temporal networks, we start by analyzing the lead time obtained from simulating SI epidemic model. The main steps are summarized as follows. First, we divide the duration of a temporal network into two consecutive time windows [0, 3*T*/4] and [3*T*/4, *T*]. Second, at time 3*T*/4, we create a surveillance set *υ* which is composed of sentinels (because of the small node size of the *Email* network, the fraction of sentinels, *f*, ranged from 10% to 20%) based on different surveillance strategies in the time window [0, 3*T*/4]. Third, at time 3*T*/4, we simulate the SI epidemic model 10 times to calculate the average lead time *ω*_*υ*_, where the infection probability in the epidemic model is 100%. In Fig. 4, we plot the performance of the average lead time *ω*_*υ*_ as a function of the fraction *f* of the individuals monitored for each surveillance strategy and for each temporal network. We find the *Recent*, *Frequent* and *Acquaintance* surveillance strategies yield similar results for the average lead time *ω*_*υ*_ on the *Prostitution* network, *Dating* network and the synthetic network (we calculate the average lead time for five temporal networks generated by the *Synthetic* model). In the SI text, we plot the distribution of the lead time.  All these three strategies provide significant average lead time to react to infectious diseases. For example, when *f* = 10%, the average lead time is about 20 days, 1.5 × 10^5^ seconds and 13 time steps respectively. Though the decrease of *ω*_*υ*_ is more gradual, the trend is that it saturates when *f* > 10%. For example, the average lead time is about 14 days, 1.35 × 10^5^ seconds and 8 time steps separately when *f* = 20%.

The *Recent*, *Frequent* and *Acquaintance* surveillance strategies can provide a substantial lead time on the email network when the surveillance set size is small. For example, the *Recent* strategy can provide approximately 706 seconds of lead time. The *Acquaintance* and *Frequent* strategies can provide approximately 1157 and 2119 seconds of lead time separately when *f* = 10%.

The *Random* strategy, which does not exploit any structure, shows the lowest efficacy on the four temporal networks.

**Figure 5:**
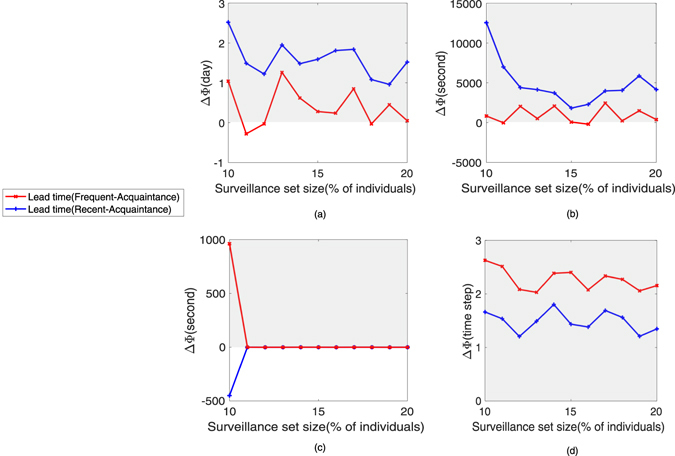
The performance of the *Recent* and *Frequent* strategies relative to the *Acquaintance* strategy. The performance measure ΔΦ is the relative average lead time. The shadow regions indicate an improvement on *Acquaintance* (the more positive values, the better). The four panels correspond to the four datasets (**a**) *Prostitution* network, (**b**) *Dating* network, (**c**) *Email* network, (**d**) *Synthetic* network.

To quantify the relative benefits of the strategies (*Recent*, *Frequent*), we plot the increase of average lead time with respect to the *Acquaintance* strategy, ΔΦ (Fig. 5). For example, ΔΦ = 10 if the strategy in question increases the average lead time by 10 time steps relative to *Acquaintance* strategy. For the *Prostitution* and *Dating* networks, the relative advantage of the *Recent* strategy is strongest. *Frequent* is similar to or better than *Acquaintance* strategy. For the *Email* network, the relative advantage of *Frequent* strategy is strongest when the surveillance set size is small. For the *Synthetic* network, the relative advantage of the *Frequent* strategy is strongest, followed by the *Recent* strategy. We use two artificial temporal networks to further investigate the effects of the temporal structure on surveillance strategies (see SI text).

We conclude that the *Recent* and *Frequent* surveillance strategies both provide a beneficial lead time for early detection of infectious diseases on a network with a strong temporal structure. Moreover, the small *f* can provide the good effect. With *f* increasing, more inactive individuals or individuals with activity in the distant future may be selected as sentinels. Thus it decreases the lead time.

### Effects of temporal correlations

In a temporal network, the structure in time can impact the dynamical processes in the same way as network structure can^33^. In order to explore the effect of temporal correlations, we consider using a temporal null model—Randomly permuted times (RP)—to remove correlations between consecutive contacts in original temporal networks. In the RP model, we fix the network’s static topological structures and the numbers of contacts between all pairs of individuals and only reshuffle the contact time stamp randomly^15, 33^. The main steps are summarized as follows: First, for each contact between a pair of individuals, we reallocate a random time stamp *τ* with uniform probability. In this method, we get the new list of contact events (*i*, *j*, *t*) describing the contact between *i* and *j* at time *t*. Second, if a contact (*i*, *j*, *t*) appears one or more times, we reallocate the random time *τ* based on the non-overlapping criterion in the randomization. Third, we apply the same surveillance strategies to this uncorrelated temporal network. Here we mainly focus on two classes of empirical datasets where the *Prostitution* and *Dating* networks are similar (we let *Prostitution* network represent this class of datasets) and the *E*-*mail* data shows a unique behavior.

**Figure 6:**
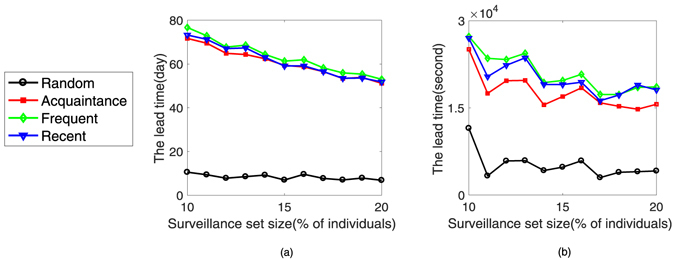
The size of surveillance set impacts performance on two randomized temporal networks. The lead time as function of the fraction of sentinels for the (**a**) randomized *Prostitution* network and (**b**) randomized *Email* network. The colors for the surveillance strategies are the same as in Fig. 4.

Figure 6 shows the performance of the average lead time *ω*_*υ*_ as a function of the fraction *f* of the population monitored for each surveillance strategy and for each type of randomized network (averaged over five generated networks). On the randomized *Prostitution* network, the curves in Fig. 6(a) are similar to Fig. 4(a,b). Only the lead time based on the randomized *Prostitution* network is longer than that based on the original network. For example, when *f* = 20%, the average lead time (the *Recent*, *Frequent* and the *Acquaintance* surveillance strategies) is about 52 days, whereas the average lead time is about 14 days on the *Prostitution* network.

On the other hand, for the randomized *E*-*mail* network, the curves in Fig. 6(b) are different from those in Fig. 4(c). The strategies (the *Recent*, *Frequent* and *Acquaintance* surveillance strategies) provide amount of lead time. For example, the average lead time is at best about 2.20 × 10^4^ seconds.

We find the changes of the persistence and the burstiness of these two randomized temporal networks are small. The size of the epidemic increases when the temporal correlations are removed from the original networks. In this situation, the chance of selecting a highly active individual is higher with the *Recent* and *Frequent* strategies. From the above analyses, we conclude that the *Recent*, *Frequent* and *Acquaintance* surveillance strategies all also provide a significant lead time on a network where the temporal correlations are removed. Furthermore, we see that the temporal correlations presented in the original datasets limit the dynamics of epidemic transmission^39^.

### Effects of the infection probability of disease

**Figure 7:**
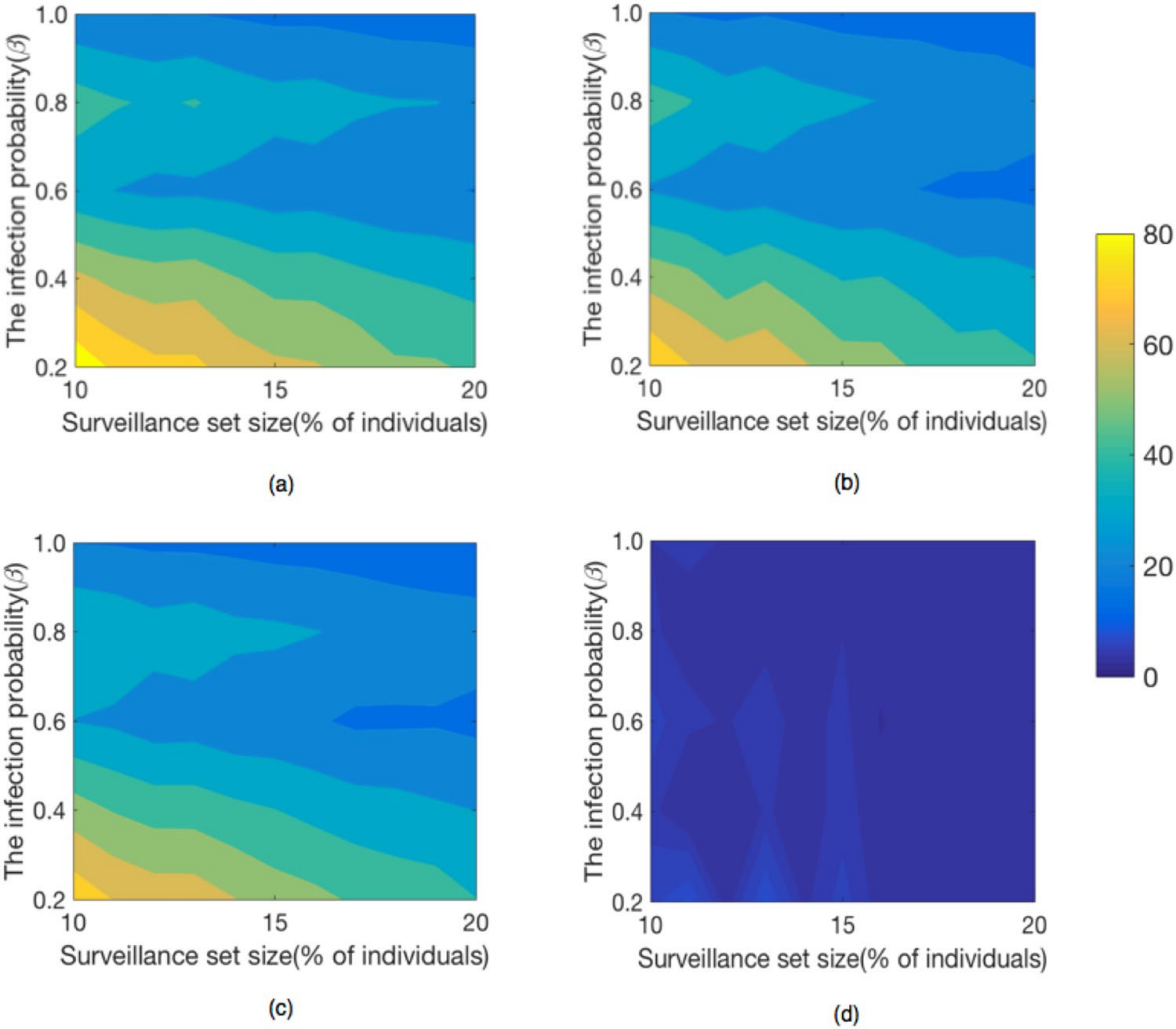
The size of surveillance set and the infection probability impact performance on *Prostitution* network. The infection lead time improves as the surveillance system expands in the (**a**) *Recent*, (**b**) *Frequent*, (**c**) *Acquaintance* and (**d**) *Random* strategies.

**Figure 8:**
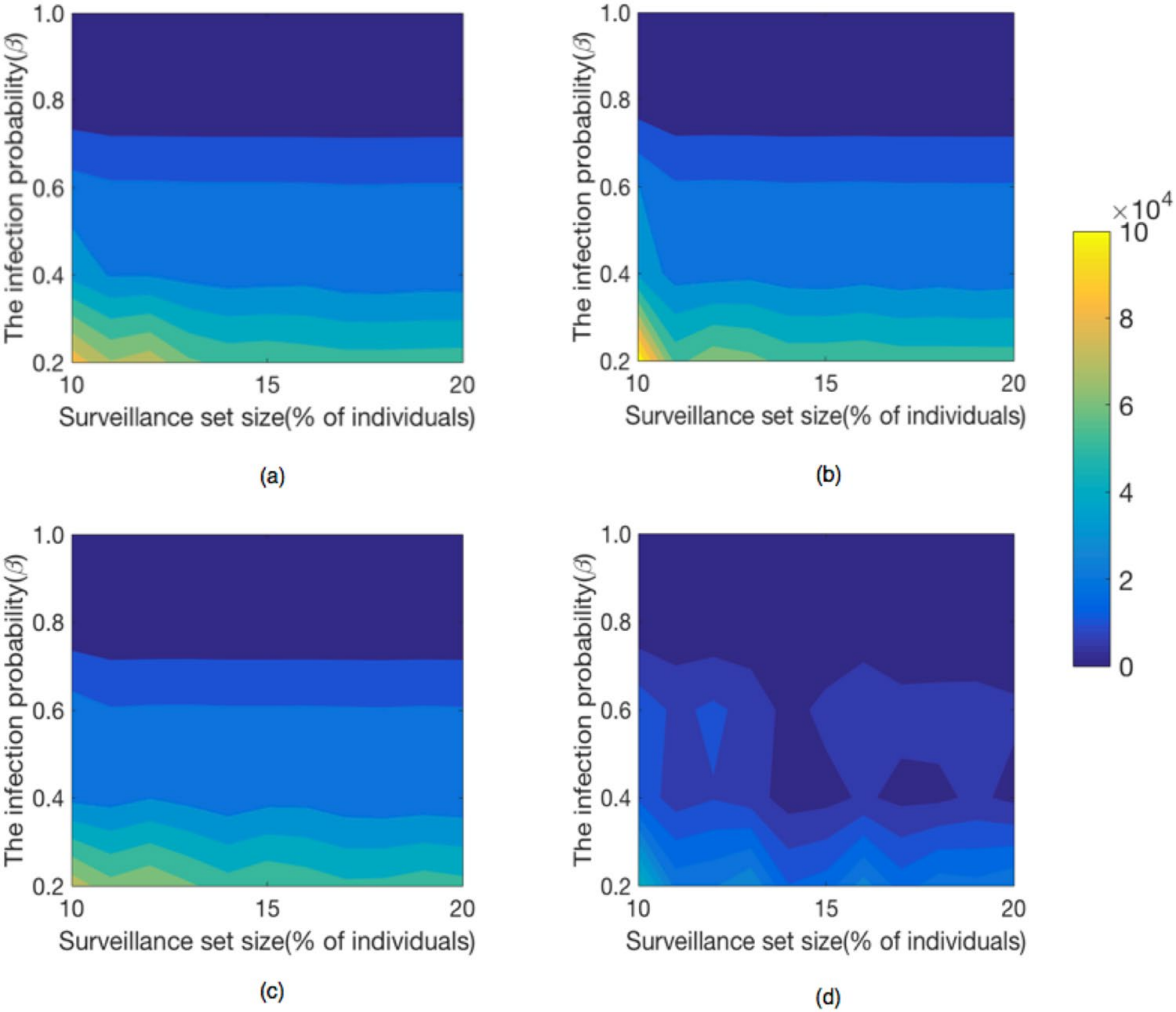
The size of surveillance set and the infection probability impact performance on the *E*-*mail* network. The infection lead time improves as the surveillance system expands in the (**a**) *Recent*, (**b**) *Frequent*, (**c**) *Acquaintance* and (**d**) *Random* strategies.

To investigate the robustness of our results, we observe the response of the average lead time *ω*_*υ*_ to the fraction of surveillance individuals *f* and the disease’s per-contact infection probability *β*. We continue to focus on *Prostitution* network and *E*-*mail* network. The main steps of selecting surveillance sentinels and simulating epidemic transmission are almost the same to those discussed above. The only difference is that we run the SI model 10 times with different *β*-values different from one. In Figs 7 and 8, we plot the variation of the average lead time *ω*_*υ*_ with the fraction *f* of the population monitored and the disease’s infection probability *β* on *Prostitution* and *Email* networks. We find that the amount of lead time depends on the fraction *f* and the disease’s infection probability *β*. For example, when *f* and *β* are small, the lead time is large. This demonstrates the small infection probability can slow down the speed of epidemic transmission and prolong the duration of the outbreak.

We find that when *f* and *β* are small, the relative advantage of *Recent* strategy is strongest on *Prostitution* network and the relative advantage of *Frequent* strategy is strongest on *Email* network.

We conclude that when the infectious disease is mild with respect to infection probability, we can sample a small fraction of individuals as sentinels exploiting temporal network structures to early detect the onset of infectious diseases.

## Conclusion

To design surveillance systems for the early detection of infectious disease outbreak it is important to optimize the set of surveillance sentinels. The understanding of the network structure of individual contacts can help health policy makers to design the optimal surveillance strategy. The contact networks not only have topological structure but also temporal structure. We found that the two temporal-network strategies *Recent* and *Frequent* and the *Acquaintance* strategy provide significant lead time, especially when the network has a very dynamic nature. Further, the *Recent* is the best strategy for the *Prostitution* and *Dating* empirical temporal networks, whereas the *Frequent* is the better strategy for the *Email* and the *Synthetic* networks. In addition, the *Recent*, *Frequent* and the *Acquaintance* strategy provide a greater amount of lead time when the infectious disease is mild. Obtaining this result will help health policy makers design more efficient surveillance systems for the early detection of infectious disease in reality.

To further understand the effect of temporal structure and parameters in an epidemic model, we investigate the network’s temporal correlations and the disease’s infection probability. Specifically, we evaluate two temporal-network strategies and two static-network strategies on two representative networks (*Prostitution* network and *E*-*mail* network). The results show the amount of lead time depends on the temporal structure of networks and the disease’s infection probability. Specifically, the two temporal-network strategies and the *Acquaintance* strategy provide a greater amount of lead time on a network where the temporal correlations are removed. On the other hand, these three strategies provide a greater amount of lead time when the disease’s infection probability is lower.

This study is a first step effort to analyze the efficiency of the surveillance strategies based on temporal structures of contact networks for the early detection of infectious disease. This study leaves room for future investigations. For example, the factors need to be investigated to well explain the early detection phenomenon on the network where temporal correlations are removed. How the burstiness of the temporal networks affect the performance of the early detection. We mainly focus on whether the two vaccination strategies on temporal networks can serve as surveillance strategies. We will study more surveillance strategies and the relationship to temporal structures.

## Electronic supplementary material

Supplementary Information
