# Supplementary material for: Optimizing sentinel surveillance in temporal network epidemiology
Source: Sci Rep. 2017 Jul 6;7:4804. doi: 10.1038/s41598-017-03868-6 (PMC5500503; doi:10.1038/s41598-017-03868-6)
Supplement: Supplementary file 1 — Supplementary Information [file 41598_2017_3868_MOESM1_ESM.pdf]

Optimizing sentinel surveillance in temporal network epidemiology

Supporting Information

Yuan Bai[1,2], Bo Yang[1,2], Lijuan Lin[1,2], Jose L. Herrera[3,4], Zhanwei Du[3], Petter Holme[5]

[1] *College of Computer Science and Technology, Jilin University, Changchun, 130012, China*

[2] *Key Laboratory of Symbolic Computation and Knowledge Engineering of Ministry of Education, Jilin University, Changchun, 130012, China*

[3] *Department of Integrative Biology, University of Texas at Austin, Austin, 78705, United States*

[4] *ICTP South American Institute for Fundamental Research, Sao Paulo State University, Sao Paulo, 03001-000, Brazil*

[5] *Institute of Innovative Research, Tokyo Institute of Technology, Tokyo, 152-8550, Japan*

SI Text

Measuring burstiness

Burstiness is used to describe the phenomenon that human activities often happen in intense periods separated by periods of quiescence. Ref. [1, 2, 3] define a measure of burstiness based on the standard deviation and the mean of the inter-contact times. Specifically, $B = (\sigma_\tau - m_\tau)/(\sigma_\tau + m_\tau)$, where σ_τ is the the standard deviation and the m_τ is the mean of the inter-contact times τ. B is bound by -1 and 1, where -1 represents a periodic sequence, 0 represents a sequence with Poissonian inter-contact time and 1 represents a maximal bursty sequence.

The distribution of the lead time

Fig. S1 corresponds to Fig.4 in the main text. Thus, Fig. S1 shows the distribution of the lead time ω_υ as a function of the fraction f of the individuals monitored for each surveillance strategy and for each temporal network.

Surveillance strategies evaluation in two artificial temporal networks

In order to further investigate the effects of the temporal structure on surveillance strategies, we use two artificial temporal networks. These two temporal networks share the same topological structure. To generate these networks, we use the following approach: First, we generate a scale free random network with a power law degree distribution [4]. Then we add time stamps for each edge in this scale free random network based on the varying activity model (the VA model) and the partner turnover model (the PT model) separately [1]. In the PT model, the consecutive time step Δt equals to 500. Since this two models are stochastic, we generate five temporal networks with each method. The dataset includes $N = 20,000$ nodes and the average $E = 1,167,400$ and spans $T = 2000$ time steps in the VA model. Correspondingly, the dataset includes $N = 20,000$ nodes and the average $E = 12,592,134$ and spans $T = 2000$ time steps in the PT model. Then we test the performance of the four surveillance strategies in these two classes of temporal networks. Here, the infection probability of the disease is 100 % and the fraction of sentinels f range from 1 % to 20 %.

In Fig. S2, we plot the performance of the early detection from the simulations of the two artificial temporal networks. For the VA model, the *Frequent* strategy works best in our range of parameters, because the change of selecting a highly active node with the *Frequent* strategy is higher. In the same way, for the PT model, the *Recent* strategy works best, because the change of selecting a highly active node with the *Recent* strategy is higher.

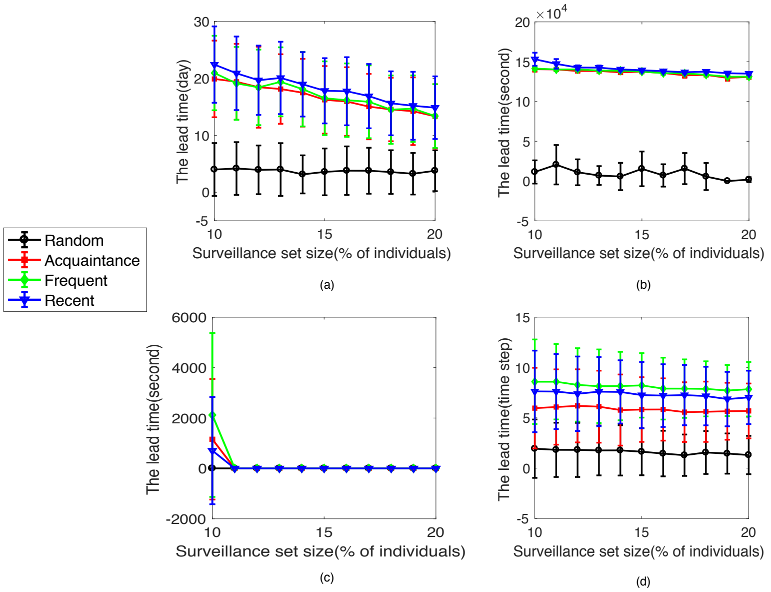

Figure S1: **The size of surveillance set impacts performance on four temporal networks in the main text.** How the lead time improves as the surveillance set expands in the (a) *Prostitution* network, (b) *Dating* network, (c) *Email* network and (d) *Synthetic* network. The surveillance set was chosen by the *Random* (black), *Acquaintance* (red), *Frequent* (green) and *Recent* (blue) strategies. Points and the error bars indicate mean and the standard deviation in performance over 100 simulations.

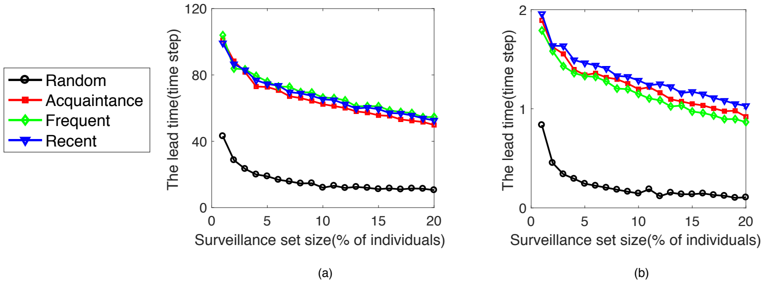

Figure S2: **The size of surveillance set impacts performance on two artificial temporal networks .** How the lead time improves as the surveillance set expands in the (a) VA network model, (b) PT network model. The surveillance set was chosen by the *Random* (black), *Acquaintance* (red), *Frequent* (green) and *Recent* (blue) strategies.

References

[1] S. Lee, L. E. Rocha, F. Liljeros, P. Holme, Exploiting temporal network structures of human interaction to effectively immunize populations, PLoS ONE 7 (5) (2012) e36439.

[2] P. Holme, Temporal networks, Springer, 2014.

[3] K.-I. Goh, A.-L. Barabási, Burstiness and memory in complex systems, EPL (Europhysics Letters) 81 (4) (2008) 48002.

[4] A.-L. Barabási, R. Albert, Emergence of scaling in random networks, science 286 (5439) (1999) 509–512.
